# Nutritional and Socioeconomic Determinants of Overweight and Obesity in the French Amazon: The Health Barometer Study

**DOI:** 10.3389/fendo.2022.849718

**Published:** 2022-04-12

**Authors:** Mickael Massicard, Kinan Drak Alsibai, Mathieu Nacher, Nadia Sabbah

**Affiliations:** ^1^Department of Endocrinology and Metabolic Diseases, Centre Hospitalier André Rosemon, Cayenne, French Guiana; ^2^Department of Pathology, Cayenne Hospital Center Andrée Rosemon, Cayenne, French Guiana; ^3^Center of Biological Resources (CRB Amazonie), Cayenne Hospital Center Andrée Rosemon, Cayenne, French Guiana; ^4^Clinical Investigation Center, Centre Hospitalier André Rosemon, University Antilles French Guiana (CIC INSERM 1424), Cayenne, French Guiana

**Keywords:** overweight, obesity, nutritional status, socioeconomic status, French Guiana

## Abstract

**Objectives:**

French Guiana is a multicultural overseas territory where obesity is a major public health problem. This study aimed to highlight the nutritional and socioeconomic determinants of overweight and obesity in different populations in French Guiana.

**Methods:**

A two-stage random sample of 1390 individuals aged 15 to 75 years was surveyed by telephone, and the participants were initially screened for diabetes. Logistic regression was fitted on the sample to adjust for potential confounding factors.

**Results:**

Overweight and obesity were found in 54.7% of the respondents, a higher proportion than in mainland France. There was a significant body image discrepancy in our population, with a higher risk of obesity among single women, often immigrants from the non-French Caribbean and South America, unemployed or low education.

**Conclusions:**

The main factors associated with obesity were being a precariousness immigrant; there was often a mismatch between body image and overweight/obesity, which is a major obstacle to the improvement of dietary behaviors and lifestyle. This information provides operational clues as to where to act and the necessary adaptations to attempt to modify behaviors in a culturally-adapted manner.

## Introduction

French Guiana is the largest French territory, with a population of 260,000 within a land area of 86500 km2. Majority of the population resides on the coast. French Guiana is divided into 2 zones: an area mostly covered by primary forest with restricted access (airplanes, canoes) where 15% of the population lives and a coastal area where lie the 3 main cities Cayenne, Saint Laurent du Maroni, and Kourou. Given its history and geography, the result of successive migration waves and the continuous immigration from neighboring countries the Guianese population is multicultural and multilingual: Over 20 languages are spoken (1). Medical care and prevention campaigns are complicated by social deprivation, the inability to read or speak the language, and different cultural representations (2).

Overweight and obesity are a major public health issue, with its global prevalence increasing from 27% to 47% in the past 30 years. More than 1.9 billion and 609 million adults worldwide are overweight or obese, respectively and this represents near 40% of the world’s population (3). The prevalence of overweight and obesity varies between regions. Obesity is a multifactorial condition influenced by genetic predisposition, sedentary lifestyle, increased food intake, energy imbalance, and psychological and socioeconomic factors (4). Obesity has important consequences at the individual level –through metabolic and cardiovascular diseases, and mechanical complications— which weigh heavily on health expenditure (5, 6). In French Guiana, the first cause of mortality is cardiovascular diseases (7) and in particular strokes linked to cardiovascular risk factors such as hypertension, overweight/obesity and diabetes (8). There are major health inequality between French Guiana and mainland France but also within French Guiana between the socially deprived and the others (9, 10). Due to insufficient infrastructures in the territory, lack of transport and geographical isolation there is also significant renouncement to, and interruption of, care. This is most salient for chronic and torpid problems: weight issues, for example.

In French Guiana, the PODIUM survey found that obesity was prevalent in 6.4% of children, 13% of men, and 22% of women (11). However, the survey was conducted in 2008, and currently, there are limited data on overweight and obesity in French Guiana. It is important to have accurate data on overweight and obesity in French Guiana to target most at risk populations and to adapt preventive measures and patient management. Thus, this study aimed to estimate the incidence of obesity and overweight in French Guiana and to highlight their nutritional and socioeconomic determinants.

## Methods

The 2014 Health Barometer survey was conducted between April and November 2014. The survey used telephone and computer assistance and employed a two-stage random sample: sampling of telephone numbers and sampling of a single respondent from among those eligible using a telephone number. First, landline and cell phone numbers were randomly generated. Then, one person living in the household was randomly selected from the eligible individuals. To be eligible, a household had to include at least one person aged between 15 and 75 years, residing in French Guiana, and speaking French or Creole. Confidentiality and anonymity were guaranteed by a procedure that erased the telephone number. The refusal rate among those contacted by telephone was lower than in Hexagonal France (9% vs. 25%), but a greater proportion of telephone numbers remained unreachable (39% vs. 18%).

The participation rate was 49%. The average duration of the questionnaire was 37 minutes. The data were weighted using sampling weights, taking into account the probability of drawing the phone number, the number of eligible persons in the household, and the number of phone lines in the household. It was then calibrated with reference data from the 2011 French Guiana population census by the National Institute of Statistics. This calibration took into account age, sex, education level, and household structure. The sample consisted of 2015 individuals aged between 15 and 75 years.

In this study, we focused on overweight and obesity as measured by body mass index (BMI) and included participants aged 18-75 years. Weight and height were given over the phone by the participant.

### Ethics Approval and Inform Consent to Participate

This study was approved by the French regulatory authority [Commission Nationale de l’Informatique et des Libertés (CNIL)], and further information on the survey methodology of the Health Barometer has been published elsewhere (12). This was a national survey and participants responded to the survey by agreeing to the use of the survey data. Among the variables studied were the standard variables used for metropolitan France (tobacco, alcohol, access to screening, access to care, mental health) supplemented by local themes (diabetes, dietary habits, vector-borne diseases, leptospirosis, and vaccinations). The following variables of interest, identified according to the literature, were also studied: sociodemographic variables (lifestyle: single, couple, or divorced, housing, place of birth, spoken language, diplomas, professional activity, socio-professional categories, monthly income, financial comfort), health care utilization, subjective perceptions, and dietary habits. Informed consent in compliance with The Declaration of Helsinki (13).

### Statistical Analysis

We first performed a descriptive analysis that explored the sample, and then the distribution of the different variables was compared between overweight or obese individuals and those with normal weight. Multivariate analysis was used to adjust for potential confounders. Bivariate analyses were performed using Pearson’s chi-square test for data weighted using the second order Rao-Scott correction. Significant variables in the bivariate analysis (i.e., those with p values <0.05) were entered into the model (the potential confounders can be seen in **Appendix 1**, **2**).

The weight and height of the patient was asked during the telephone interview in order to calculate the BMI.

## Results

### Participant Characteristics

**Figure 1:**
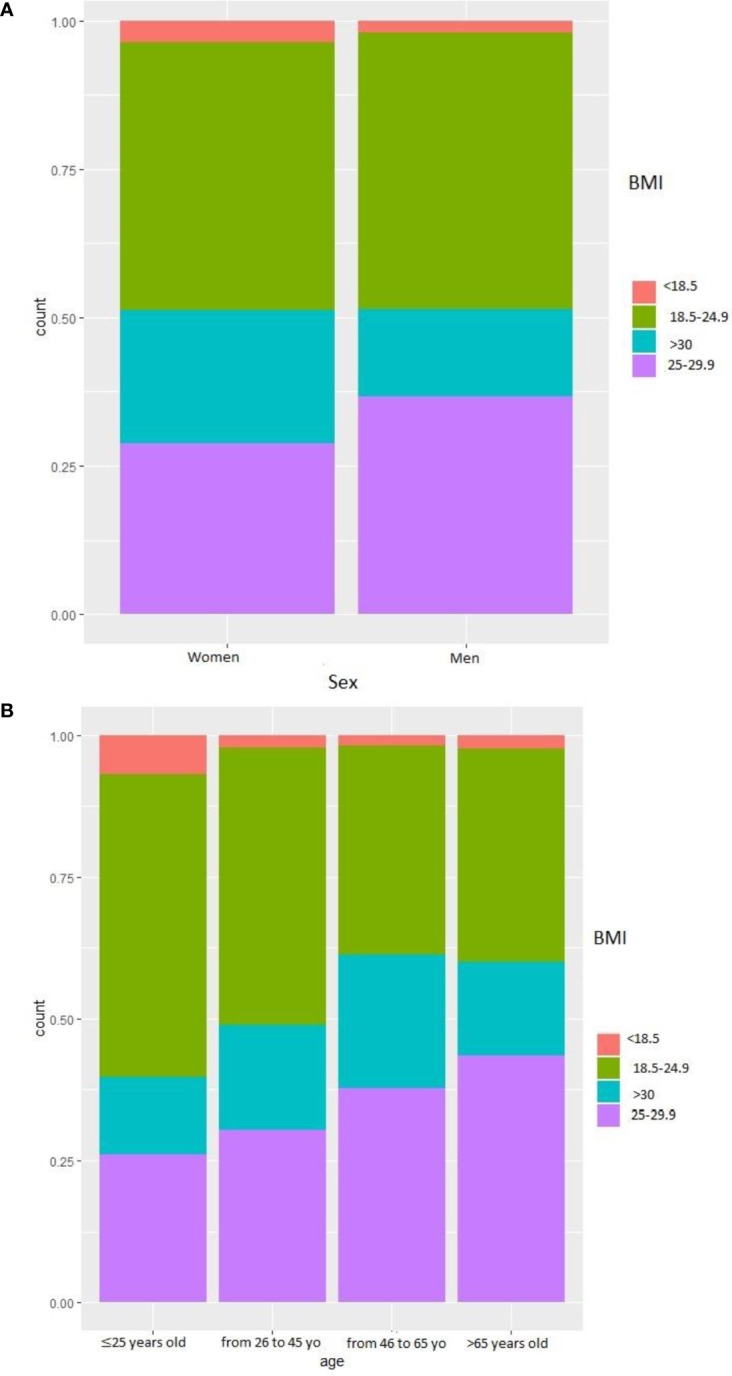
BMI of the participants. **(A)** BMI according to sex. **(B)** BMI according to age.

The average participant age was 38.7 years. The mean weight was 74.1 kg, and the mean BMI was 26.22 kg/m^2^. Anthropometric data and age are described in **Table 1**. More than half of the population was overweight or obese [54.7% (95% CI: 51.7-58)]. Specifically, 18.8% (95% CI: 16.7-21) and 35.9% (95% CI: 33-39) of the participants were obese and overweight, respectively. The sex distribution of male and female participants was equal, and 50% of the participants declared themselves as living in a couple. Overall, 7.5% of the study population had self-reported diabetes, and 20% reported having smoked at least once in their lives, 11.7% of whom reported smoking daily. The proportion of overweight/obese participants did not differ according to sex, but women were more affected by obesity than men (**Figure 1**).

**Table 1:** Description of weighted quantified factors.

Variables	Mean	Standard deviation	Minimum	Maximum	First quartile	Median	Third quartile
**Age**	38.70	13,91	18	75	27	37	48
**Weight**	74.10	15,44	35	195	64	73	83
**Height**	168.24	10,42	120	195	161	168	175
**BMI**	26.22	5,34	13,67	59,17	22.97	25.4	28.69

**Figure 2:**
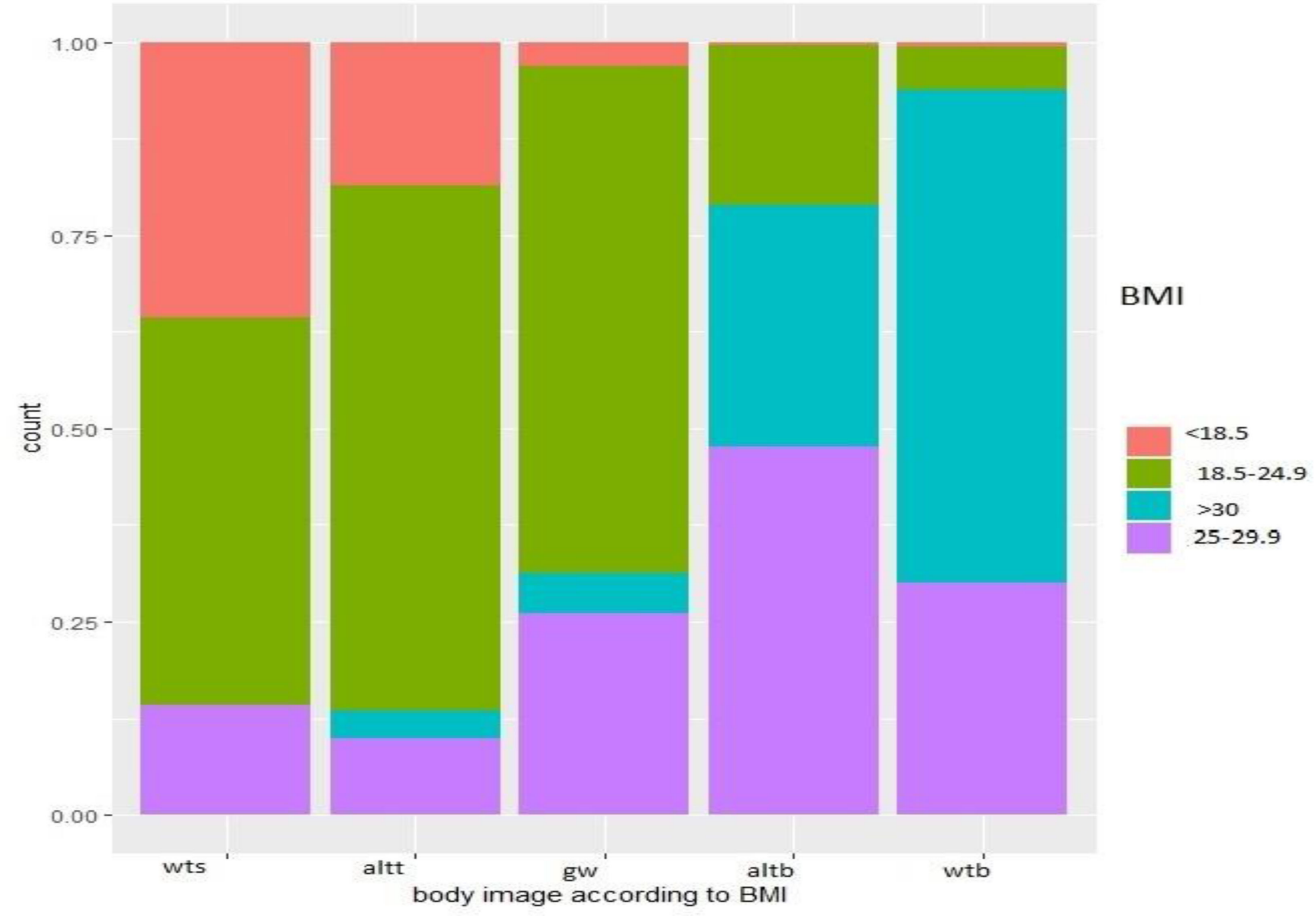
Body image according to BMI. Wts, way too skinny; altt, a little too thin; Gw, quite the good weight; Altb, a little too big; Wtb, way too big.

The rate of overweight increased with age, but obesity was more prevalent in the 45-65 age group than in the over-65 age group (**Figure 1**). Comparison of the answers between the participant’s perception of their weight (“much too thin”, “a little too thin”, “about the right weight”, “a little too fat”, “much too fat”) and the actual weight showed that a significant proportion of overweight or obese individuals thought they were “much too skinny”, “a little too skinny,” or “about the right weight”. Similarly, a significant proportion of obese people considered themselves “a little too fat” (**Figure 2**).

Bivariate analysis about general characteristics can be seen in **Appendix 1**.

### Nutritional Characteristics and Socioeconomic Characteristics

**Figure 3:**
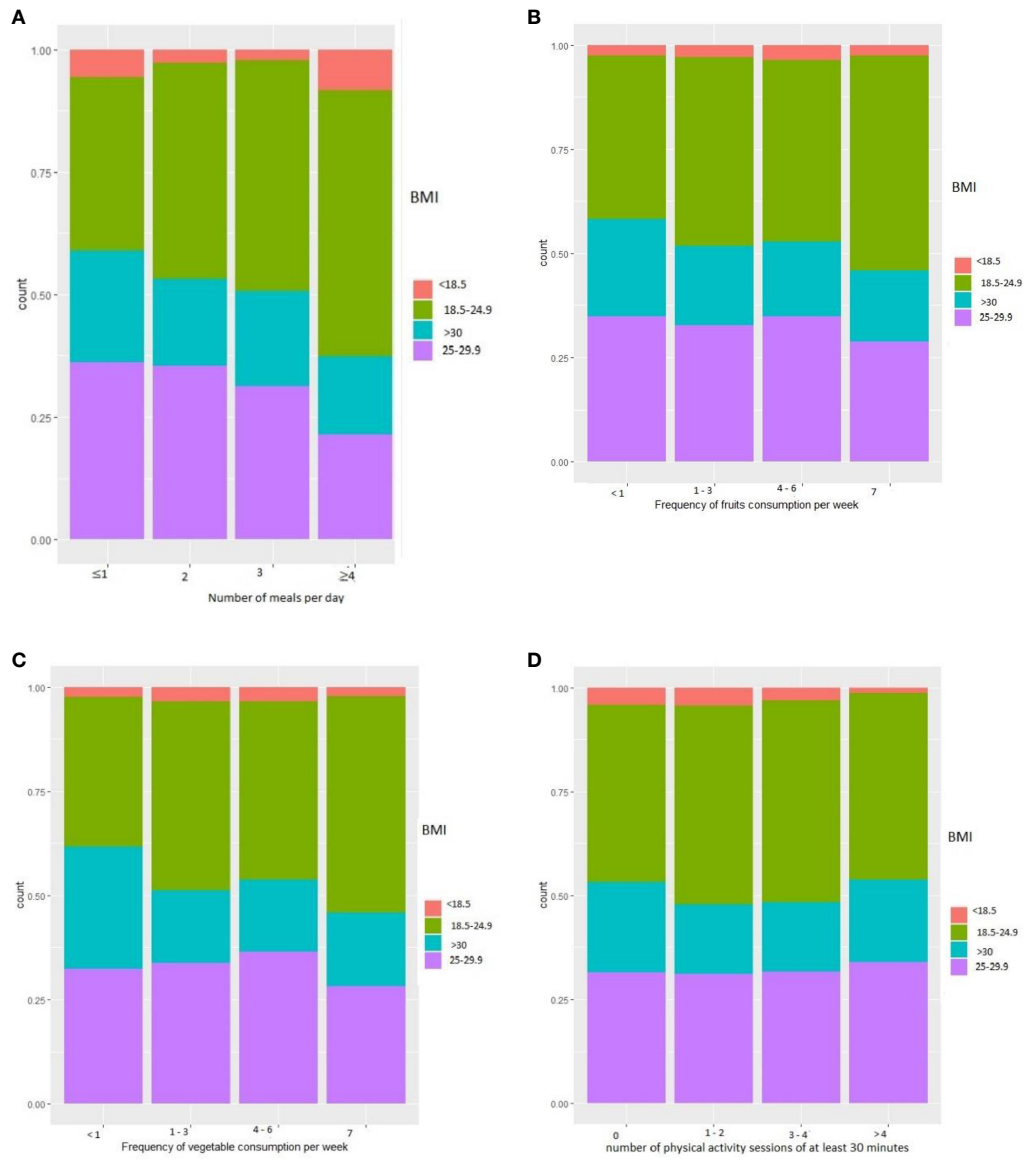
Nutritional characteristics. **(A)** BMI according to the frequency of meals per day. **(B)** BMI according to the frequency of fruit consumption per week. **(C)** BMI according to the frequency of vegetable consumption per week. **(D)** BMI according to the number of physical activity sessions of at least 30 minutes.

Analysis of the number of meals eaten per day to body weight showed a higher proportion of participants with overweight and obesity among those eating ≤1 and 2 meals per day than among those eating 3 meals per day (**Figure 3**). Those with daily fruit consumption were less overweight or obese than those consuming fruit less than once daily (**Figure 3**). Those who consumed vegetables daily were less corpulent than those who consumed them less than once a week (**Figure 3**). For physical activity, 22.7% reported 30 minutes of physical activity (work, travel, leisure) every day, while 24% were completely sedentary. There was minimal difference in body size by level of weekly physical activity (**Figure 3**). More than 33% of the population reported consuming sweetened beverages at least 4 times per week, 5.8% consumed wine more than 3 times per week (50%consumed wine ≤1 time per week), and 13% consumed beer several times per week (50% consumed beer ≤1 time per week).

Socioeconomic characteristics are described in **Tables 2**, **3**.

**Table 2:** Description of binary socioeconomic factors.

Living as a couple	50.2%
Asset Solidarity Income [RSA :Revenue de Solidarité Active]	15.6%
Supplemental health insurance	21.8%

**Table 3:** Description of binary socioeconomic factors.

Housing							
House		Appartementsinblocks		Privateappartement		«Carbet»	
**60%**		**26.5%**		**9.4%**		**3.3%**	
**Place of birth**							
French Guiana	Caribbean (except French WestIndies)		SouthAmerica	MainlandFrance		French overseas territories	Others
48,7%	17.1%		14.6%	11.3%		5.5%	2.7%
**Spoken language**							
French	French lexified creole	Portuguese	English-lexifiedcreole	Others	English	AmerIndians	Spanish
55%	22%	8.9%	7.6%	2.4%	1.6%	1.3%	1.2%
**Diplomas**							
Noneor9th grade		2yearsafterthe8thgradeandthe9thgrade		Hight school degree and 1oneat4yearsafter	Master degreeandmore	Don’tknow	
40.8%		28%		23.7%	6.3%	1.2%	
**Professionnal activity**							
Employed	Unemployed		Stayedathome		Student	Re-retirementandretirement	Other
53.3%	23.3%		8.4%		7.7%	6%	1.1%
**Socio-professional categories**							
Employees		Unknown		Workers	Technicians	Managers	
41.5%		27.7%		17%	8.6%	7.6%	
**Monthly income**							
Not known	Lessthan1000euros		1000-1500euros	1500-2000euros	2000-3000euros	3000-4500euros	Morethan4500euros
12.4%	15.8%		18.2%	16.2%	21.9%	8.8%	6.7%
**Financial comfort**							
NotKnown		«Confortable»		«it’sok»	«it’sshort»	«It’sdifficult»	«withdebts»
0.4%		15.8%		36.7%	20.5%	20.7%	5.9%

Carbet, small informal wooden house open to the outside without water or electricity.

Bivariate analysis about nutritional characteristics can be seen in **Appendix 2**.

### Independent Factors of Obesity and Overweight

Being in a relationship, having the highest household income, having diabetes, having a current or past history of smoking, having benefited from the minimum insertion income (aid which aims to guarantee a minimum level of resources and facilitate the insertion or reintegration of people with low incomes) in the past year, professional degree, diploma, place of birth, beer consumption, vegetable consumption, number of meals per day, number of dinners taken per week, socio-professional category, financial comfort, net monthly income per household, and age were significantly different between normal or lean and overweight or obese individuals in the bivariate analysis and were thus included in the logistic regression model (**Tables 4**, **5**). Meanwhile, sex, fruit consumption, sweetened beverage consumption, number of breakfasts and lunches eaten per week, and weekly frequency of physical activity of at least 30 minutes were not significant (**Appendix 1**, **2**).

**Table 4:** Logistic regression results (OR>1).

Variables	OR	IC95%
Being in a relationship	1.36	1.011.84
Have the highest household income	1.08	0.80 1.45
Being diabetic	2.44	1.40 4.25
Have benefited from RSA last year	1.27	0.83 1.93
Have no diploma or 9^th^ grade	2.46	1.42 4.27
Have High school degree	1.13	0.59 2.17
Have professional High school degree	2.19	1.10 4.36
2 years after the 9^th^ grade	1.68	0.92 3.07
2 years after the 8^th^ grade	1.22	0.70 2.10
Place of birth « other »	1.24	0.56 2.72
Place of birth: French overseas departments	1.10	0.59 2.04
Place of birth: French Guiana	1.02	0.64 1.63
Consumption of vegetables 4 to 6 times per week	1.39	0.96 2.02
Consumption of vegetables never or less than once a week	1.43	0.90 2.28
Daily Vegetable Consumption	1.11	0.77 1.59
Never dine	1.76	0.71 4.35
Dinner less than once a week	1.17	0.50 2.78
Financial comfort: “it’s okay”	1.18	0.80 1.74
Financial comfort: “it’s short”	1.46	0.95 2.23
Financial comfort: “not without running into debt”	1.57	0.75 3.31
Financial comfort: “it’s difficult”	1.75	1.08 2.83
Age from 46 to 65 years	1.20	0.86 1.67
Age ≤ 25 years	1.15	0.73 1.79
Fruit consumption 4 to 6 times per week	1.18	0.80 1.75

OR, Odd Ratio; RSA, Asset Solidarity Income.

**Table 5:** Logistic regression results (OR<1).

Variables	OR	IC95%
Being or having been a smoker	0.68	0.48 0.96
Professional status «other »	0.55	0.16 1.90
Being unemployed	0.62	0.41 0.93
Being a student	0.22	0.11 0.44
Stay at home	0.45	0.25 0.82
Being retired or pre-retired	0.90	0.44 1.82
3 years after Hight school degree	0.82	0.46 1.48
Master degree and more	0.67	0.38 1.18
Technologic High school degree	0.93	0.42 2.06
Place of birth: Caribbean except French West Indies	0.84	0.48 1.48
Place of birth; mainland France	0.72	0.40 1.27
Eating 2 meals a day	0.86	0.49 1.52
Eating 3 meals a day	0.95	0.53 1.67
Eating 4 or more meals a day	0.43	0.20 0.93
Dinner 4 to 6 times a week	0.69	0.38 1.27
Dinner every day	0.76	0.47 1.23
Age over 65years	0.60	0.24 1.48
Fruit consumption per week never or less than once	0.94	0.63 1.41
fruit consumption; every day	0.85	0.59 1.22
Consumption of sugary drinks 4 to 6 times per week	0.82	0.53 1.29
Never consume sugary drinks	0.97	0.60 1.57
Consumption of sugary drinks less than once a week	0.73	0.49 1.09
consumption of sugary drinks: every day	0.85	0.60 1.20

## Discussion

Despite their profound health and economic impact, overweight and obesity are not sufficiently studied in French Guiana. In this survey, 54.7% of the participants were overweight or obese (overweight, 35.9%; obese,18.8%) and obesity was more prevalent in the 45-65 age group than in the over-65 age group., a significant proportion of obese people considered themselves “a little too fat”. Women were more frequently affected by obesity. People who were overweight or obese were more often those who consumed vegetables the least frequently. Risk factors associated with obesity included low education, immigration, and diabetes. In France, overweight and obesity are prevalent in 49.3% and 17.2% of individuals, respectively (14). In neighboring Brazil, the prevalence rate of obesity in 2014 was 20.7% (15), and the prevalence of overweight was as high as 70% in the poorest neighborhoods in the north (16). The prevalence of obesity in the Latin American adult populations has high variability, ranging from 9.9% up to 35% (17). In Mexico City, the prevalence of overweight and obesity among women is over 73%, the prevalence of obesity among latin american women living in the united states is over 40% (18). In chili we noticed the prevalence of obesity was 18.1% of men and 27.5% of women (19). A study realized in Surinam showed 22% of obesity in 2016, and a higher risk of becoming obese when we are a woman and being married (20).

Comparison between body size and body image showed that a significant proportion of people with a normal weight or overweight considered themselves “a little too thin”, while people who were obese considered themselves “a little too fat”. Although, there may be a degree rationalization to reduce cognitive dissonance, this mostly raises important issues on the cultural perceptions of overweight and obesity in the Guianese population. According to the health belief model, if persons do not recognize an issue as a problem, aiming to change their behavior will be a challenging enterprise; Furthermore, if social norms do not perceive overweight as pathological or esthetically less beautiful, social cognitive theory would also suggest changing eating behaviors might also be difficult (21, 22). A 2014 study in the French West Indies showed that out of 10 overweight participants, 4 participants perceived themselves as having a normal body weight. Meanwhile, 6 out of 10 patients claimed that no health professional ever told them they were overweight before (23).

Medical information on obesity can improve the patient’s perception of his or her obesity, but this information is not provided in French Guiana due to the very low number of nutritional specialists (24). In this study, women belonging to the Creole community were more likely to underestimate their body weight. One study showed that women of black and Hispanic ethnicity only reported body image discrepancy when they were overweight (25). In some cultures, being overweight is a sign of fertility in women and can also be a canon of beauty (26). In black South African community, being overweight, regardless of sex, is seen as a way of affirming important social status (26).

Women tended to be more obese or overweight than men. Notably, the proportion of obese participants was higher among women than among men. In contrast, overweight tended to be more prevalent in men, but the difference was not significant. The higher proportion of obese women can be explained by pregnancies. The fertility rate in 2014 was 2.56 (27), and pregnancy increases the risk of obesity. In addition, there are many single-parent families in French Guiana. These parents raise several children alone with little time for self-care and to perform physical activities. In the context of increasing obesity prevalence, there are nevertheless widespread micronutrient deficiencies in pregnant women, which have long been overlooked and are often associated with obesity (28). Associations between micronutrient deficiencies and obesity have been reported in various populations, and such deficiencies may affect leptin and insulin metabolisms. Micronutrient deficiency –hidden hunger—may impair appetite regulation and energy metabolism and some intervention studies have shown that correcting them was associated with appetite regulation (29).

Another important factor influencing weight is physical activity (30). In this study, there was an equal proportion of participants who reported having and not having daily physical activity. Thus, we were unable to assess the influence of physical activity on body weight. This is probably because of the lack of a detailed assessment on the type of activity performed and the intensity of these activities.

The multivariate analysis identified being in a relationship, having diabetes, and being a smoker were independent factors associated with overweight and obesity.

In French Guiana, food insecurity is associated with a greater probability of obesity among those living with partners, married women, and widows than in never-married women (31). The higher risk of being overweight among individuals in a relationship can be explained by the inducement to eat (32). At the socioeconomic level, just under half of the population was born in French Guiana. Nearly 17% of the respondents were from the non-French Caribbean territories, and 14.8% were from South America, highlighting the importance of immigration in our territory. More than a third of our population had no diplomas, and 23% were unemployed. The socio-professional categories were essentially represented by employees and workers. Smoking is known to have an appetite suppressant effect, and nicotine increases resting energy expenditure (33). Only half of the population ate 3 meals a day and breakfast was the most often missed meal, with 11.2% of the population never eating breakfast. Not eating breakfast has been associated with a higher risk of overweight and obesity (34, 35).

In the multivariate analysis, only eating 4 or more meals per day was significant and seemed to have a protective against being overweight. However, information on the nutritional quality of meals and their proportions were not available. Participants who reported eating more than 4 times a day probably eat better quality meals and in lower quantity than those who ate 3 meals a day. The timing of food intake can also affect weight regulation (36). In addition, food intake frequency is inversely associated with BMI, with eating frequency having an inverse relationship with body weight (37).

In total, 28%and 30.9% of the participants reported consuming fruit and vegetables once a day, respectively, thus falling far short of the recommendation to eat at least 5 fruits and vegetables per day. Weekly vegetable consumption was a significantly factor in bivariate analysis but not in multivariate analysis consistent with to the financial difficulties that lead patients to buy cheaper –often highly caloric— products than vegetables, which are very expensive in French Guiana (10). We could not assess the impact of the consumption of sweetened beverages on obesity because the type of sweetened beverages and its quantification could not be realized. More than a quarter of the population surveyed consumed sweetened beverages daily. Increased consumption of sweetened beverages is associated with an increase in body size. Increased consumption of sweetened beverages during childhood or adolescence could even predict weight gain in adulthood (38).

The proportion of overweight or obese people was significantly higher in those from the non-French Caribbean and South American nations, those who received the Solidarity Income (Revenu de Solidarité Active, whichensures a minimum level of income for people without resources)in the past year, those who were physically active, those with no diploma, those who were employed, considering that financially “it’s difficult”, having a net monthly household income between 600 and 2400 euros. Meanwhile, the proportion of people of normal weight individuals was higher in those with a net monthly household income of <600 euros or >2400 euros.

Multivariate analysis identified having no diploma and having a vocational high-school degree to consider that financially “it is difficult”. Being unemployed, a student, or a homemaker was associated with a lower risk of being overweight or obese. The lower risk of being overweight among homemakers could be explained by the possibility of having more physical activity from housework (more time preparing meals) and being more economically comfortable. A Canadian study showed that a high level of education was protective against the risk of obesity. Further, immigrant status was inversely associated with the risk of obesity, in contrast with the trend in our study. However, it should be noted that migrant populations differ between Canada and French Guiana. In a recent study realized in one hundred seventy-six Latin American cities within eight countries (Brazil, Chile, Colombia, Costa Rica, El Salvador, Guatemala, Mexico and Peru) we observed protective from obesity for women whereas for men it was only if the city had a high level of development (39).

The limitations of this study include the lack of precise assessments on the nutritional quality of meals and the proportion of food consumed and the lack of a concise definition of physical activity. The study was limited to French and Creole speaking populations, which excluded other common population groups from Brazil, Suriname, or Guiana. The weight and height being declared by the patient on the phone, it is possible that the weight is sometimes underestimated. Despite these limitations, to our best knowledge, this is the first study to explore population characteristics that may influence body weight in French Guiana. Further, this is the only second study to estimate the prevalence of overweight and obesity in French Guiana. A more detailed study on the nutritional plan and on the lifestyle of the patients with an approach on the perception of the disease and its body must be carried out in order to set up personalized strategies according to the territory, the ethical and social origins of the patients.

## Conclusion

Overweight and obesity was widespread affecting 54.7% of the study population. There was a significant body image discrepancy, with a higher risk of obesity among women, immigrants, unemployed, and with low education. The nutritional balance was poor, with little consumption of fruits and vegetables and an inadequate distribution of meals, particularly the absence of breakfast. Information on balanced nutrition and the importance of regular physical activity is necessary, and this should be targeted to the least educated and most precarious populations. Importantly, preventive measures against obesity and overweight should be adapted to each population to take in to account local representations. Finally, advising people to eat healthy food will fail if they cannot afford it; efforts should be made to reduce the costs of fresh fruits and vegetable, which remain far too expensive for the socially deprived.

## Data Availability Statement

The original contributions presented in the study are included in the article/supplementary material. Further inquiries can be directed to the corresponding author.

## Ethics Statement

The studies involving human participants were reviewed and approved by Commission Nationale de l’Informatique et des Libertés. The patients/participants provided their written informed consent to participate in this study.

## Author Contributions

Conceptualization, MN and NS. Data curation, MM, KD, and NS. Formal analysis, MN. Investigation, MM, KD, MN, and NS. Supervision, NS. Writing – original draft, MM and NS. Writing – review and editing, MM, KD, MN, and NS.

## Conflict of Interest

The authors declare that the research was conducted in the absence of any commercial or financial relationships that could be construed as a potential conflict of interest.

## Publisher’s Note

All claims expressed in this article are solely those of the authors and do not necessarily represent those of their affiliated organizations, or those of the publisher, the editors and the reviewers. Any product that may be evaluated in this article, or claim that may be made by its manufacturer, is not guaranteed or endorsed by the publisher.

## Appendix 1 Bivariate analysis general characteristics

**Table:**

Variables	p	n
Age	0.0000	1755
Sex	0.6780	1755
Leaving as a couple	0.0222	1755
Diabetes	0.0000	1754
Tabagism (Actual or not)	0.0112	1755

## Appendix 2 Bivariate analysis of nutritional characteristics

**Table:**

Variables	p	n
Number of meals per days	0.0053	1748
Number of diners per weeks	0.0074	1750
Frequency of consumption of fruits per weeks	0.4027	1748
Frequency of consumption of vegetables per weeks	0.0270	1748
Frequency of physic activities “of at least 30 min “per weeks	0.7992	1755
Frequency of consumption of sugary drinks per weeks	0.1775	1752
Frequency of consumption of wine per weeks	0.3682	1585
Frequency of consumption of beers per weeks	0.0436	1584

## References

[1] Sabbah N Carles G Demar M Nacher M. Diabetes in French Guiana, Adapting National Standards of Therapeutic Education and Care to the Amazonian Challenge. World J Diabetes (2021) 12(2):98−107. doi: 10.4239/wjd.v12.i2.98 33594330 PMC7839167

[2] Jolivet A Cadot E Florence S Lesieur S Lebas J Chauvin P. Migrant Health in French Guiana: Are Undocumented Immigrants More Vulnerable? BMC Public Health (2012) 12:53. doi: 10.1186/1471-2458-12-53 22260085 PMC3355028

[3] Chooi YC Ding C Magkos F. The Epidemiology of Obesity. Metabolism (2019) 92:6−10. doi: 10.1016/j.metabol.2018.09.005 30253139

[4] Goodarzi MO. Genetics of Obesity: What Genetic Association Studies Have Taught Us About the Biology of Obesity and its Complications. Lancet Diabetes Endocrinol (2018) 6(3):223−36. doi: 10.1016/S2213-8587(17)30200-0 28919064

[5] Kim DD Basu A. Estimating the Medical Care Costs of Obesity in the United States: Systematic Review, Meta-Analysis, and Empirical Analysis. Value Health (2016) 19(5):602−13. doi: 10.1016/j.jval.2016.02.008 27565277

[6] Tremmel M Gerdtham U-G Nilsson PM Saha S. Economic Burden of Obesity: A Systematic Literature Review. Int J Environ Res Public Health (2017) 14(4):435. doi: 10.3390/ijerph14040435 PMC5409636 28422077

[7] Rochemont DR Lemenager P Franck Y Farhasmane A Sabbah N Nacher M. The Epidemiology of Acute Coronary Syndromes in French Guiana. Ann Cardiol Angeiol (Paris) (2021) 70(1):7−12. doi: 10.1016/j.ancard.2020.09.032 33067006

[8] Rochemont DR Mimeau E Misslin-Tritsch C Papaix-Puech M Delmas E Bejot Y. The Epidemiology and Management of Stroke in French Guiana. BMC Neurol (2020) 20(1):109. doi: 10.1186/s12883-020-01650-2 32209060 PMC7093981

[9] Van Melle A Cropet C Parriault M-C Adriouch L Lamaison H Sasson F. Renouncing Care in French Guiana: The National Health Barometer Survey. BMC Health Serv Res (2019) 19:99. doi: 10.1186/s12913-019-3895-6 30728033 PMC6366016

[10] Nacher M Deungoue S Brousse P Adenis A Couppié P Sobesky M. [The Interplay Between Isolation and Precariousness, and Hospitalization Duration in French Guiana]. Rev Epidemiol Sante Publique (2020) 68(2):125−32. doi: 10.1016/j.respe.2019.09.012 32035728

[11] Daigre J-L Atallah A Boissin J-L Jean-Baptiste G Kangambega P Chevalier H. The Prevalence of Overweight and Obesity, and Distribution of Waist Circumference, in Adults and Children in the French Overseas Territories: The PODIUM Survey. Diabetes Metab (2012) 38(5):404−11. doi: 10.1016/j.diabet.2012.03.008 22626474

[12] Sabbah N Massicard M Mathieu N. Specificities of the Diabetic Population in French Guiana: The Health Barometer Survey. Curr Diabetes Rev (2021) 18(1):1–9. doi: 10.2174/1573399817666210129103506 33511949

[13] World Medical Association. World Medical Association Declaration of Helsinki: Ethical Principles for Medical Research Involving Human Subjects. JAMA (2013) 310(20):2191−4. doi: 10.1001/jama.2013.281053 24141714

[14] Matta J Carette C Rives Lange C Czernichow S. Épidémiologie De L’obésité En France Et Dans Le Monde. Presse Méd (2018) 47(5):434−8. doi: 10.1016/j.lpm.2018.03.023 29703570

[15] Martins-Silva T Vaz JDS Mola CLd Assunção MCF Tovo-Rodrigues L. Prevalence of Obesity in Rural and Urban Areas in Brazil: National Health Survey, 2013. Rev Bras Epidemiol (2019) 22:e190049. doi: 10.1590/1980-549720190049 31460664

[16] Melo SPdSd Cesse EÂP Lira PICd Ferreira LCCdN Rissin A Batista Filho M. Overweight and Obesity and Associated Factors in Adults in a Poor Urban Area of Northeastern Brazil. Rev Bras Epidemiol (2020) 23:e200036. doi: 10.1590/1980-549720200036 32428190

[17] Rivera-Andrade A Luna MA. Trends and Heterogeneity of Cardiovascular Disease and Risk Factors Across Latin American and Caribbean Countries. Prog Cardiovasc Dis (2014) 57(3):276−85. doi: 10.1016/j.pcad.2014.09.004 25218566

[18] Daviglus ML Talavera GA Avilés-Santa ML Allison M Cai J Criqui MH. Prevalence of Major Cardiovascular Risk Factors and Cardiovascular Diseases Among Hispanic/Latino Individuals of Diverse Backgrounds in the United States. JAMA (2012) 308(17):1775−84. doi: 10.1001/jama.2012.14517 23117778 PMC3777250

[19] Villanueva B Arteaga A Maiz A Cortés VA. Abdominal Obesity is a Common Finding in Normal and Overweight Subjects of Chile and is Associated With Increased Frequency of Cardiometabolic Risk Factors. PloS One (2018) 13(3):e0194644. doi: 10.1371/journal.pone.0194644 29579094 PMC5868807

[20] Khadan J Spencer N Strobl E Tuﬀour T. Factors Associated With Being Overweight or Obese in Suriname. Int J Public Health (2021) 66. doi: 10.3389/ijph.2021.1604101/full PMC8565296 34744598

[21] Saghafi-Asl M Aliasgharzadeh S Asghari-Jafarabadi M. Factors Influencing Weight Management Behavior Among College Students: An Application of the Health Belief Model. PloS One (2020) 15(2):e0228058. doi: 10.1371/journal.pone.0228058 32032376 PMC7006943

[22] Bagherniya M Taghipour A Sharma M Sahebkar A Contento IR Keshavarz SA. Obesity Intervention Programs Among Adolescents Using Social Cognitive Theory: A Systematic Literature Review. Health Educ Res (2018) 33(1):26−39. doi: 10.1093/her/cyx079 29293954

[23] Carrère P Moueza N Cornely V Atallah V Hélène-Pelage J Inamo J. Perceptions of Overweight in a Caribbean Population: The Role of Health Professionals. Fam Pract (2016) 33(6):633−8. doi: 10.1093/fampra/cmw061 27450987

[24] Mawardi G Kirkland EB Zhang J Blankinship D Heincelman ME Schreiner AD. Patient Perception of Obesity Versus Physician Documentation of Obesity: A Quality Improvement Study. Clin Obes (2019) 9(3):e12303. doi: 10.1111/cob.12303 30816010

[25] Fitzgibbon ML Blackman LR Avellone ME. The Relationship Between Body Image Discrepancy and Body Mass Index Across Ethnic Groups. Obes Res (2000) 8(8):582−9. doi: 10.1038/oby.2000.75 11156434

[26] Micklesfield LK Lambert EV Hume DJ Chantler S Pienaar PR Dickie K. Socio-Cultural, Environmental and Behavioural Determinants of Obesity in Black South African Women. Cardiovasc J Afr (2013) 24(9−10):369−75. doi: 10.5830/CVJA-2013-069 24051701 PMC3896104

[27] Leonco L Kallel H Nacher M Thelusme L Dueymes M Mhiri R. Does Universal Screening for Gestational Diabetes Mellitus Improve Neonatal Outcomes in a Socially Vulnerable Population: A Prospective Study in French Guiana. Front Endocrinol (2021) 12:644770. doi: 10.3389/fendo.2021.644770 PMC8176854 34093431

[28] Duclau A Abad F Adenis A Sabbah N Leneuve M Nacher M. Prevalence and Risk Factors for Micronutrient Deficiencies During Pregnancy in Cayenne, French Guiana. Food Nutr Res (2021) 65:5268–76. doi: 10.29219/fnr.v65.5268 PMC7955516 33776616

[29] Astrup A Bügel S. Micronutrient Deficiency in the Aetiology of Obesity. Int J Obes 2005 (2010) 34(6):947−8. doi: 10.1038/ijo.2010.81 20543852

[30] Garawi F Devries K Thorogood N Uauy R. Global Differences Between Women and Men in the Prevalence of Obesity: Is There an Association With Gender Inequality? Eur J Clin Nutr (2014) 68(10):1101−6. doi: 10.1038/ejcn.2014.86 24918120

[31] Hanson KL Sobal J Frongillo EA. Gender and Marital Status Clarify Associations Between Food Insecurity and Body Weight. J Nutr (2007) 137(6):1460−5. doi: 10.1093/jn/137.6.1460 17513407

[32] Jeffery RW Rick AM. Cross-Sectional and Longitudinal Associations Between Body Mass Index and Marriage-Related Factors. Obes Res (2002) 10(8):809–15. doi: 10.1038/oby.2002.109 12181390

[33] Bush T Lovejoy JC Deprey M Carpenter KM. The Effect of Tobacco Cessation on Weight Gain, Obesity, and Diabetes Risk. Obesity Silver Spring Md (2016) 24(9):1834−41. doi: 10.1002/oby.21582 PMC5004778 27569117

[34] Song WO Chun OK Obayashi S Cho S Chung CE. Is Consumption of Breakfast Associated With Body Mass Index in US Adults? J Am Diet Assoc (2005) 105(9):1373−82. doi: 10.1016/j.jada.2005.06.002 16129078

[35] Uzhova I Mullally D Peñalvo JL Gibney ER. Regularity of Breakfast Consumption and Diet: Insights From National Adult Nutrition Survey. Nutrients (2018) 10(11):1578. doi: 10.3390/nu10111578 PMC6267347 30373105

[36] Lopez-Minguez J Gómez-Abellán P Garaulet M. Timing of Breakfast, Lunch, and Dinner. Effects on Obesity and Metabolic Risk. Nutrients (2019) 11(11):2624. doi: 10.3390/nu11112624 PMC6893547 31684003

[37] Zhu Y Hollis JH. Associations Between Eating Frequency and Energy Intake, Energy Density, Diet Quality and Body Weight Status in Adults From the USA. Br J Nutr (2016) 115(12):2138−44. doi: 10.1017/S0007114516001112 27109636

[38] Yoshida Y Simoes EJ. Sugar-Sweetened Beverage, Obesity, and Type 2 Diabetes in Children and Adolescents: Policies, Taxation, and Programs. Curr Diabetes Rep (2018) 18(6):31. doi: 10.1007/s11892-018-1004-6 PMC6025796 29671076

[39] Mazariegos M Auchincloss AH Braverman-Bronstein A Kroker-Lobos MF Ramírez-Zea M Hessel P. Educational Inequalities in Obesity: A Multilevel Analysis of Survey Data From Cities in Latin America. Public Health Nutr (2021) 1−9. doi: 10.1017/S1368980021002457 PMC7613035 34167613

